# Analysis of Chloroplast Genome Characteristics and Codon Usage Bias of *Styphnolobium japonicum* f. *oligophyllum*

**DOI:** 10.3390/cimb48060617

**Published:** 2026-06-15

**Authors:** Zhi-Qiang Mu, Xiao Zhang, Jing-Jing Yan, Bao-Ping Chen, Hong-Wei Wang

**Affiliations:** 1 School of Architecture, Kashi University, Kashi 844000, China; mzq2023@ksu.edu.cn (Z.-Q.M.);; 2 College of Plant Protection, Henan Agricultural University, Zhengzhou 450000, China

**Keywords:** *Styphnolobium japonicum* f. *oligophyllum*, chloroplast genome, codon usage bias, optimal codons

## Abstract

To investigate the codon usage bias (CUB) and its influencing factors in the chloroplast genome of *Styphnolobium japonicum* f. *oligophyllum*, we sequenced, assembled and annotated the genome using Illumina high-throughput sequencing, and systematically analyzed 52 protein-coding sequences. The chloroplast genome is 158,739 bp with a typical quadripartite structure, containing 129 functional genes. It presents a mean GC_3_ content of 28.26% and a mean ENC value of 45.40, indicating weak CUB and low gene expression. Among 31 preferred codons (RSCU > 1), 29 (93.5%) end with A/U. Neutrality plot, ENC-plot and PR2-plot analyses reveal that natural selection is the primary regulator of CUB. A total of 19 optimal codons were identified. These results provide fundamental reference data that may facilitate future genetic engineering efforts in *S. japonicum* f. *oligophyllum*.

## 1. Introduction

Chloroplasts are essential organelles within plant cells, serving as the primary sites for photosynthesis. They possess their own genome, which encodes dozens of proteins that play critical roles in various physiological and biochemical processes of chloroplasts [1,2]. The chloroplast genome exhibits several advantageous features, including structural stability, high gene expression efficiency, a relatively small molecular size, multiple copies per cell, and amenability to efficient genetic transformation. These characteristics have facilitated its widespread application in plant identification, molecular evolution studies, genetic engineering, and phylogenetic analyses [3,4].

Among these applications, chloroplast genetic engineering—leveraging the unique properties of the plastid genome, such as maternal inheritance, absence of gene silencing, and the capacity for multi-gene co-expression—has emerged as a powerful platform for crop improvement and biopharmaceutical production [5,6,7]. In agriculture, the introduction of genes involved in photosynthetic efficiency optimization, osmotic protection, and redox regulation has significantly enhanced CO_2_ assimilation capacity and tolerance to abiotic stresses such as drought, high temperature, and salinity [4]. For instance, the accumulation of glycine betaine in tobacco effectively protects the photosynthetic apparatus from drought-induced damage [8]. Moreover, chloroplast engineering has successfully conferred resistance to insects, pathogens, and herbicides in various crops [4]. To date, plastid transformation systems have been established in tobacco, soybean, carrot, cotton, potato, and lettuce [9,10,11]. Notably, a “marker-free” one-step transformation strategy developed in potato has paved the way for commercial applications [10].

In the field of biopharmaceuticals, chloroplasts serve as efficient biofactories for the high-level expression of vaccine antigens, therapeutic proteins, and industrial enzymes [9,12,13]. For example, an antifungal enzyme cocktail (chitinase, glucanase, and mannanase) expressed in lettuce chloroplasts effectively degrades the cell wall of *Candida albicans* and inhibits fungal growth in clinical samples from oral cancer patients [11]. Additionally, a biological containment system utilizing the rare UGA codon in microalgal chloroplasts enables the safe expression of target genes that are toxic to *Escherichia coli* [14].

Despite remaining challenges, such as low transformation efficiency in some species and time-consuming homoplasmy selection [3,4], the continued development of standardized frameworks and novel transformation vectors positions chloroplast engineering to play a pivotal role in climate-resilient agriculture and sustainable biomanufacturing [3,10]. Therefore, in-depth investigation into the codon usage bias and optimal codon composition of plant chloroplast genomes is of great significance, as it will enhance the translational efficiency of chloroplast transgenes and facilitate the stable, high-level expression of heterologous genes of interest.

Codon usage bias refers to the non-random usage of different synonymous codons in species or genes [14]. Its formation is comprehensively affected by multiple factors such as genomic nucleotide composition (GC content), gene length, expression level, and environmental stress [10,13], and is the result of the dynamic balance among mutational pressure, natural selection, and genetic drift [8]. Studies have shown that CUB widely exists among different species, tissues, organs, and genes [11,15,16], and is more significant in highly expressed genes, with its intensity increasing with the elevation of gene expression level [17].

*S. japonicum* f. *oligophyllum* (Fabaceae), a form of *S*. *japonicum* in the genus Styphnolobium, is also known as butterfly pagoda tree, and *Sophora japonica oligophylla* is its synonym. Its leaflets are clustered palmately with a unique leaf shape, showing high ornamental value [18]. Meanwhile, it has the characteristics of drought tolerance, barren tolerance, and strong stress resistance, and is widely used in landscaping, building material processing, and other fields. At present, the structural characteristics of the chloroplast genome in the genus *Styphnolobium* have been reported [19,20], but research on the codon usage bias of *S. japonicum* f. *oligophyllum* remains blank. In this study, by analyzing the structural characteristics and codon usage patterns of its chloroplast genome, we aim to provide a reference for genome evolution and genetic improvement of *Styphnolobium* plants.

## 2. Materials and Methods

### 2.1. Sampling, DNA Extraction Sequencing and Annotation

In this study, fresh leaves of *S. japonicum* f. *oligophyllum* were collected in May from the Chinese Pagoda Tree Garden in Shenqiu, Henan Province, China. The plant was taxonomically identified by Hiroyoshi Ohashi (Taxonomist of Fabaceae, Tohoku University, Japan). In contrast to the genus *Sophora*, which can generally form effective symbiotic associations with rhizobia, a key physiological characteristic of the genus *Styphnolobium* is that its roots typically lose this ability. Genomic DNA was extracted and subjected to Illumina paired-end (PE) sequencing (2 × 150 bp). Using the chloroplast genome of *Styphnolobium japonicum* (GenBank accession no. MG784459) as a reference—*Sophora japonica* is a synonym of *Styphnolobium japonicum*—genome assembly was performed with SPAdes v3.15.3 [21]. The mapping rate of the raw reads to the assembled contigs was >99.5%, and the coverage uniformity across the entire chloroplast genome showed no significant drop or bias. These results confirm the robustness of our assembly and the absence of appreciable reference bias. Annotation was conducted using the online tool CPGAVAS2, followed by manual correction with Geneious v9.0 [22]. The complete chloroplast genome sequence of *Styphnolobium japonicum* f. *oligophyllum* obtained in this study was deposited in the National Center for Biotechnology Information (NCBI) under the accession number ON571618.

### 2.2. Calculation of Codon Nucleotide Composition and Preference Parameters

Short coding DNA sequences (CDSs) tend to cause large deviations in codon usage analysis; thus, CDSs shorter than 300 bp were excluded prior to calculation. Two short plastid genes, *ycf15* and *ycf68*, were discarded following this screening criterion due to their insufficient sequence length. Protein-coding sequences from the annotated chloroplast genome of *S. japonicum* f. *oligophyllum* were manually extracted using Geneious R9.0.2. Redundant sequences and sequences shorter than 300 bp were removed, and 52 CDSs were ultimately retained for codon usage bias analysis. The 52 qualified CDSs were combined into a single FASTA file. Codon composition, codon adaptation index (CAI), codon bias index (CBI), and frequency of optimal codons (Fop) were determined using CodonW 1.4.2. The CUSP program in the EMBOSS online tool (Version 6.6.0) was used to analyze codon preferences, including GC content at the first (GC_1_), second (GC_2_), and third (GC_3_) codon positions, as well as the mean GC content across all three positions (GC_all_). Statistical analysis was performed with SPSS 22.0.

### 2.3. Analysis of Relative Synonymous Codon Usage

The relative usage of synonymous codons was analyzed as the ratio of the observed usage frequency to the theoretical usage frequency of codons in the chloroplast genome of *S. japonicum* f. *oligophyllum*. The relative synonymous codon usage (RSCU) values of the 52 CDSs were calculated. An RSCU value of 1 indicates no codon usage bias; an RSCU value > 1 indicates higher usage frequency of the codon; otherwise, the codon usage frequency is lower [23].

### 2.4. Neutrality Plot Analysis

A neutrality plot can be used to analyze factors affecting codon usage bias [24]. A scatter plot was generated with GC_3_ as the abscissa and GC_12_ (GC_12_ = (GC_1_ + GC_2_)/2) as the ordinate; each point on the plot represents one gene. If the scatter points are uniformly distributed along the diagonal line, the regression coefficient of the standard curve is close to 1, suggesting no significant difference between GC_12_ and GC_3_ and no obvious divergence in base-pair composition at the three codon positions. Under this circumstance, the gene is less affected by selection pressure and more prone to mutational bias. Conversely, when the scatter points deviate from the diagonal, the standard regression coefficient approaches zero, indicating a remarkable difference between GC_12_ and GC_3_, and the codon usage of this gene is mainly shaped by selection pressure.

### 2.5. ENC-Plot Analysis

The effective number of codons (ENC) is also an important indicator for analyzing codon usage bias [24,25], and its value can be used to identify highly and lowly expressed genes. ENC-plot analysis was performed with ENC as the ordinate and GC content at the third codon position (GC_3_) as the abscissa. The plot contains a standard curve and scatter points, where each circle represents one protein-coding gene.

### 2.6. PR2-Plot Bias Analysis

The A, T, G, and C content at the third position of each codon in the chloroplast genome of *S. japonicum* f. *oligophyllum* were first determined. Then, a Parity rule 2 plot (PR2-plot) scatter diagram was constructed with the proportion of G at the third position relative to G + C (G_3_/(C_3_ + G_3_)) as the abscissa and the proportion of A at the third position relative to A + T (A_3_/(T_3_ + A_3_)) as the ordinate. The central reference point (0.5, 0.5) represents complete base equilibrium (A = T, G = C), which is the neutral state without bias. The direction and degree of bias of other genes are indicated by the vector distance between this point and the central reference point [26].

### 2.7. Determination of Optimal Codons

Sequences were sorted by the effective number of codons (ENC) values, and the top and bottom 10% of sequences at both ends were selected to construct the high-expression library and low-expression library, respectively. The ΔRSCU values of the gene were then calculated. A codon was defined as an optimal codon if it met two criteria: ΔRSCU ≥ 0.08 (high-expression codon) and RSCU > 1 (high-frequency codon) [27].

## 3. Results

### 3.1. Characteristics of the Chloroplast Genome of S. japonicum f. oligophyllum

**Figure 1:**
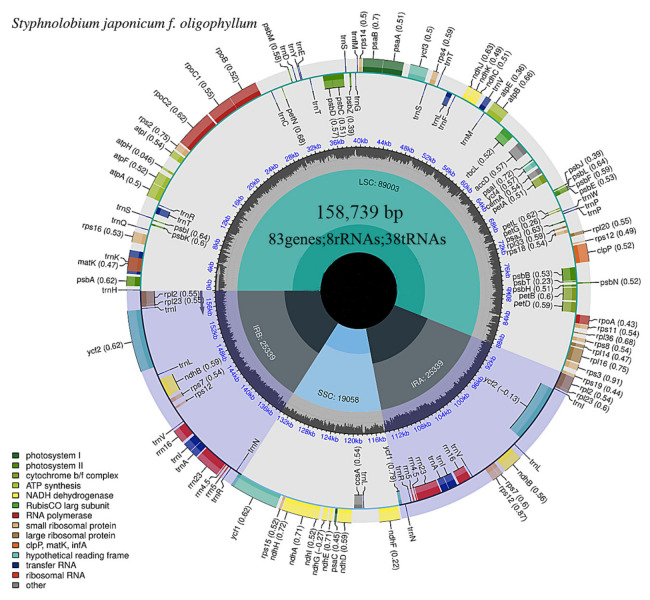
Circular map of the complete chloroplast genome of *S. japonicum* f. *oligophyllum*.

The sequencing results showed that the complete chloroplast genome of *S. japonicum* f. *oligophyllum* was 158,739 bp in length, exhibiting a typical quadripartite structure of angiosperm chloroplast genomes (Figure 1), with distinct characteristics in each structural partition. The total GC content of this chloroplast genome was 36%, and significant differences in GC content were detected among different structural regions: the large single-copy (LSC) region had a GC content of 33.5%, the small single-copy (SSC) region had the lowest GC content at only 29.61%, and the inverted repeat (IR) region had the highest GC content, reaching 43.15%. This distribution pattern was highly consistent with the GC content characteristics of most angiosperm chloroplast genomes.

Gene composition analysis indicated that the chloroplast genome of *S. japonicum* f. *oligophyllum* had an identical gene composition to that of *S. japonicum* (accession number: MG784459). Both genomes contained 129 unique functional genes, which fell into three major categories: 83 protein-coding genes (PCGs), 38 tRNA genes, and 8 rRNA genes. According to the basic biological functions of the genes, the functional genes in the chloroplast genome of *S. japonicum* f. *oligophyllum* could be divided into four categories, as detailed in Table 1.

Further analysis of gene copy number characteristics revealed that the genome contained 18 duplicated genes, each with two copies. These genes belonged to three categories: seven protein-coding genes, namely *ycf2*, *ycf*1, *rps12*, *rps7*, *rpl23*, *rpl2*, and *ndhB*; four rRNA genes, namely *rrn23S*, *rrn16S*, *rrn5S*, and *rrn4.5S*; seven tRNA genes, namely *trnA-UGC*, *trnI-CAU*, *trnI-GAU*, *trnL-CAA*, *trnN-GUU*, *trnR-ACG*, and *trnV-GAC*.

### 3.2. Codon Composition and Preference Parameter Analysis

Analysis was conducted on 52 CDS sequences from the chloroplast genome of *S. japonicum* f. *oligophyllum*. The results are shown in (Table 2). The average contents of GC_1_, GC_2_, GC_3_ and GC_all_ were 46.56%, 39.37%, 28.26% and 38.06%, respectively. Among them, the GC_2_ content was relatively close to the overall level of GC_all_, while the difference between GC_1_ and GC_3_ contents was significant. The GC contents at all three codon positions were lower than 50%, and showed an overall decreasing pattern of GC_1_ > GC_2_ > GC_3_, with obvious differences in nucleotide composition among different positions.

Further analysis of the variation range of GC content at each codon position revealed distinct differences in the extreme GC content values among different genes. GC_1_ content ranged from 31.79% to 58.38%, with the highest value in *clpP* and the lowest in *ccsA*; GC_2_ content ranged from 33.72% to 53.24%, with the highest value in *rps11* and the lowest in *ycf1*; GC_3_ content ranged from 21.69% to 36.81%, with the highest value in *ycf2* and the lowest in *ndhF*; GC_all_ content ranged from 29.14% to 45.10%, with the highest value in *rps12* and the lowest in *ycf1.* Combined with the GC content distribution characteristics at the above positions, it can be confirmed that the chloroplast genome of *S. japonicum* f. *oligophyllum* prefers codons ending in A or U, and the third codon position shows a stronger bias toward A or U.

The ENC values of the codons ranged from 36.63 to 52.65 (Table 2), with an average of 45.40, indicating a weak codon usage bias. The CAI values ranged from 0.1 to 0.29 (average 0.17); the CBI values ranged from −0.22 to 0.17 (average −0.10), reflecting that the usage frequency of optimal codons was lower than the average usage frequency of all codons.

The Fop values ranged from 0.27 to 0.52 (average 0.35), indicating that the proportion of optimal codons among synonymous codons was small. The above results demonstrate a weak codon usage bias in the chloroplast genome of *S. japonicum* f. *oligophyllum*.

**Figure 2:**
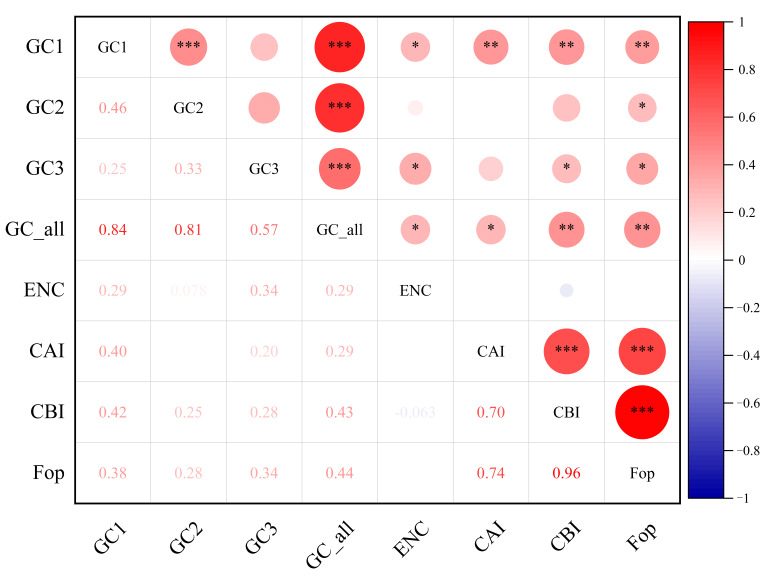
Correlation Analysis of Parameters in the Genome. Note: *** *p* < 0.001,** *p* < 0.01, * *p* < 0.05.

GC_1_, GC_2_ and GC_3_ were all highly significantly correlated with GC_all_ (*p* < 0.001) (Figure 2). GC_1_ exhibited a highly significant positive correlation with GC_2_, whereas GC_3_ showed only weak correlation with GC_1_ and GC_2_. This indicated that the base composition and usage at the first and second codon positions were similar, but distinct from those at the third position. ENC was significantly positively correlated with GC_1_, GC_3_ and GC_all_ (*p* < 0.05), but showed little correlation with GC_2_, implying that the GC content at the second codon position had little effect on codon usage bias. GC_1_ showed extremely significant positive correlations with CAI, CBI and Fop (*p* < 0.01); GC_2_ was significantly positively correlated with Fop; GC_3_ was significantly positively correlated with CBI and Fop; GC_all_ was highly significantly positively correlated with CBI and Fop, and significantly positively correlated with CAI. These results indicated that base composition was closely associated with codon usage bias in the chloroplast genome of *S. japonicum* f. *oligophyllum*.

### 3.3. Results of Relative Synonymous Codon Usage Analysis

**Figure 3:**
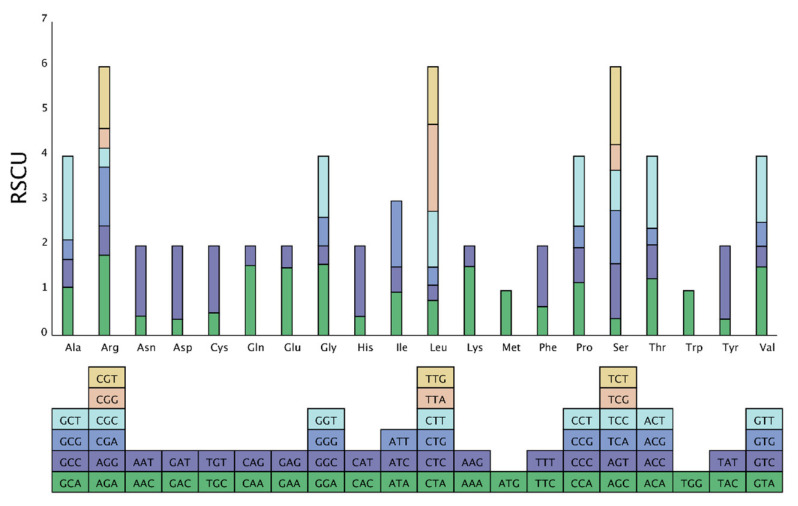
RSCU of codon in chloroplast genome.

According to RSCU data analysis (Figure 3), 31 codons in *S. japonicum* f. *oligophyllum* had an RSCU > 1. Of these, 13 (41.9%) ended with A, 16 (51.6%) with U, 1 (3.25%) with G, and 1 (3.25%) with C. The proportion of codons ending in A + U reached 93.5%, indicating a strong preference for A/U-ending codons in its chloroplast genome.

### 3.4. Results of Neutrality Plot Analysis

**Figure 4:**
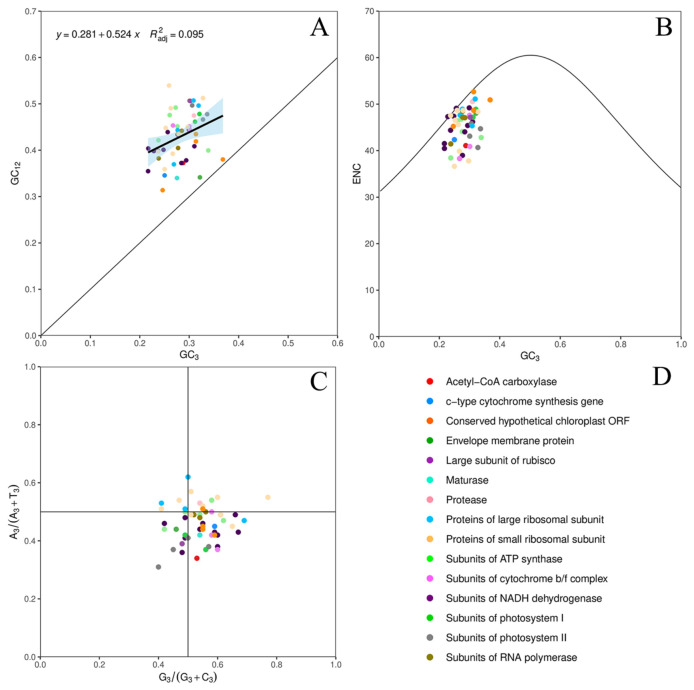
Plots of the causes of codon preference in the chloroplast genome. (**A**) Neutrality plot analysis; (**B**) ENC plot analysis; (**C**) PR2-plot analysis; (**D**) Functional classification legend for chloroplast genome proteins/genes.

GC_12_ (the average of GC_1_ and GC_2_) ranged from 31% to 54%, while GC_3_ varied narrowly from 22% to 37%, showing the typical AT bias of chloroplast genomes (Figure 4A). The first and second codon positions are under stronger functional constraints for amino acid coding, whereas the third synonymous site exhibited a more distinct AT preference. The correlation coefficient (R^2^) between GC_12_ and GC_3_ was 0.095, and the regression slope (y = 0.524) was significantly less than 1, indicating that codon usage bias in the chloroplast genome was mainly driven by natural selection rather than mutation pressure.

### 3.5. Results of ENC-Plot Analysis

The ENC values of all chloroplast genes of *S. japonicum* f. *oligophyllum* were significantly lower than the theoretical maximum of 61, indicating an obvious codon usage bias in its chloroplast genome (Figure 4B). All gene points were concentrated in the low GC range of 22–37% for GC_3_, and lay significantly below the standard curve with only a few genes near the curve. This distribution pattern suggests that natural selection plays an important role in codon usage bias. Notably, deviations from the theoretical curve can also be caused by multiple factors including amino acid composition constraints, so mutation pressure is unlikely to act as the sole driving force.

Among the 52 protein-coding genes in the chloroplast genome of *S. japonicum* f. *oligophyllum*, 77% had ENC ratios ranging from −0.05 to 0.15 (40 genes) (Table 3). Only 23% showed a deviation > 0.15, and just one gene (2%) fell in the high-deviation range of 0.25–0.35. This indicates that the observed ENC values of most genes differed slightly from the theoretical values predicted by GC_3_, suggesting a relatively weak effect of mutation pressure. Combined with the ENC-plot analysis, we infer that codon usage bias is jointly shaped by multiple forces, in which natural selection exerts a notable influence.

### 3.6. Results of PR2-Plot Bias Analysis

PR2-plot analysis showed that the third codon base usage bias of all protein-coding genes was moderate (Figure 4C). All gene points clustered around the central reference point (0.5, 0.5) without extreme aggregation in a single quadrant. G_3_/(G_3_ + C_3_) was mainly distributed at 0.4–0.6 (slightly > 0.5), indicating a slightly higher G than C content; A3/(A3 + T3) ranged from 0.3 to 0.5 (slightly < 0.5), showing a slightly higher T than A content, which is consistent with the typical AT bias of chloroplast genomes. PR2-plot mainly reflects the characteristics of base compositional bias. Since complementary evidence such as dN/dS ratios and gene expression data is not available in this study, we do not further infer relevant selective mechanisms. The overall distribution pattern is generally consistent with the distribution features revealed by neutrality plot and ENC-plot.

### 3.7. Results of Optimal Codon Determination

There are a total of 22 high-expression codons with ∆RSCU ≥ 0.08 (Table S2). Among these codons, 19 have an RSCU value greater than 1 and are identified as optimal codons, namely *GCU*, *CGU*, *AAU*, *GAU*, *UGU*, *CAA*, *GAA*, *GGU*, *CAU*, *AUU*, *UUA*, *AAA*, *UUU*, *CCC*, *AGU*, *UCU, ACU*, *GUA*, and *GUU*. Among these optimal codons, 13 ended with U, 5 ended with A, and 1 ended with C, indicating a strong preference for codons ending in A/U.

## 4. Discussion

The chloroplast genome of *S. japonicum* f. *oligophyllum* presents a typical circular quadripartite structure. Its core characteristics, including genome length, structural partitioning, GC content and gene composition, are highly consistent with previous studies on intraspecific taxa of *Styphnolobium japonicum* (syn. *Sophora japonica*) [19,28,29], further verifying the relatively conserved evolution of its chloroplast genome. Such structural conservation is widespread across *Sophora* and the segregated genus *Styphnolobium*; most species within these two genera possess a typical quadripartite plastid architecture with a steady genome size ranging from 151,270 bp to 158,837 bp [20,30,31], while *Sophora xanthantha* possesses an obviously larger plastome (172,376 bp), primarily resulting from substantial expansion of the IR region [31]. The GC content varies significantly across different structural regions, following the pattern IR regions > LSC region > SSC region. This pattern has been confirmed in closely related taxa [20,28,30,32]. IR regions have a high GC content, which may be related to the enrichment of abundant RNA-coding genes and higher sequence conservation in the IR regions.

Numerous studies have confirmed that codon optimization can effectively improve the expression efficiency of plant target genes to a certain extent [27,31,33]. Analysis of the chloroplast genome of *S. japonicum* f. *oligophyllum* in this study showed that the GC content differed among different codon positions, with an average order of GC_1_ > GC_2_ > GC_3_, meaning the GC content at the first two positions of codons was higher than that at the third position. This pattern is consistent with the results of codon preference studies in *Macadamia integrifolia* [23], *Actinostemma tenerum* [34], *Sphaerophysa salsula* [35], *Koelreuteria bipinnata* [36] and other plant species, as well as in closely related *Sophora* taxa [32]. In this study, 31 synonymous codons with RSCU > 1 were identified in the chloroplast genome of *S. japonicum* f. *oligophyllum*, and 93.5% of the high-frequency codons ended with A + U. A total of 19 optimal codons were detected, among which 94.74% ended with A/U, demonstrating the core characteristics of strong AT bias, predominant U-ending, and formation mainly driven by natural selection. This AT bias preference is consistent with findings for *Sophora tonkinensis* and *Sophora alopecuroides* [32,37]. This further confirms that the codon structure of the chloroplast genome is relatively conserved in higher plants during evolution, with high similarity in codon usage patterns. Meanwhile, some differences (Table S3) exist in the type and number of optimal codons among different plants, suggesting that distinct plant lineages have experienced different selective pressures during evolution.

Notably, several genes deviate from the overall AT-biased codon usage. Genes *psbA*, *psbB*, *psbC*, and *psbD* exhibit GC_1_ > 50%, likely due to functional constraints, as they encode core Photosystem II proteins (D1, CP43, CP47, D2) essential for photosynthesis. Elevated GC_1_ favors hydrophobic amino acids, facilitating membrane protein folding and stability. Similarly, *clpP* shows exceptionally high GC_1_ (58.38%), possibly required for encoding a conserved protease domain or specific RNA secondary structures for post-transcriptional regulation. In contrast, *ccsA* and *rps18* display low GC_1_, which may enhance translation efficiency in the AT-rich chloroplast genome by better matching the AT-biased tRNA pool. These examples suggest that natural selection dominates codon usage bias, but its direction and intensity are modulated by individual gene functions. Further functional validation is needed to clarify these differential selective pressures.

In this study, neutrality plot, ENC-plot and PR2-plot were comprehensively applied to analyze the formation mechanism of codon usage bias in its chloroplast genome. Neutrality plot analysis showed that the correlation coefficient between GC_12_ and GC_3_ was extremely low (R^2^ = 0.095), and the regression slope (0.524) was significantly less than 1. This indicated that the first and second codon positions were under strong functional constraints, the third codon position showed more significant AT bias, and codon usage bias was mainly dominated by natural selection. This dominant role of natural selection is consistent with results in Sophora and related genera [37]. In the ENC-plot analysis, the ENC values of all genes were lower than the theoretical maximum, and gene points were concentrated in the low GC_3_ range (22–37%) and distributed below the standard curve. The ENC ratio distribution showed that 77% of the genes had ratios concentrated in the range of −0.05 to 0.15 with small deviations, further confirming that codon usage bias was dominated by natural selection, while mutation pressure played only a weak role. It is worth noting that ENC is only an indirect analytical indicator for gene identification and codon usage bias evaluation. Relying solely on ENC values cannot accurately identify gene characteristics, and auxiliary verification combined with other analytical indicators is essential to obtain credible conclusions. PR2-plot analysis revealed that gene points were evenly distributed around the central reference point (0.5, 0.5). G_3_/(G_3_ + C_3_) was slightly higher than 0.5, and A_3_/(A_3_ + T_3_) was slightly lower than 0.5, showing a trend that G was slightly more abundant than C and T was slightly more abundant than A, which was consistent with the typical AT bias characteristic. These results suggested that base usage at the third codon position was shaped by both natural selection and mutation pressure, and natural selection effectively restricted extreme bias. The above patterns are consistent with the findings of closely related *Sophora* species, including *Sophora alopecuroides* [32] and *Sophora tonkinensis* [37]. In contrast, the codon usage bias in species such as *Sphaerophysa salsula* [35] is jointly shaped by natural selection and mutation pressure, indicating that the dominant driving factors differ among species.

Notably, under overall strong AT-biased mutational pressure across the chloroplast genome, distinct GC divergence at the first codon position is observed among functional genes: *psbA*/*psbB*/*psbC*/*psbD* exceed 50% in GC_1_, *clpP* possesses an extremely high GC_1_ value of 58.38%, while ccsA and rps18 show unusually low GC_1_. The intrinsic causes behind such heterogeneous GC distribution remain unclear with current available data, which deserves further multi-omics and transgenic verification in future studies.

## 5. Conclusions

This study systematically analyzed the structural characteristics and codon usage bias pattern of the chloroplast genome of *S. japonicum* f. *oligophyllum*. It was clarified that the chloroplast genome of this species exhibits high evolutionary conservation in sequence composition, structural arrangement and gene constitution, and natural selection was the dominant driving force underlying codon usage bias. These findings provide a theoretical basis for dissecting the molecular evolutionary mechanism, optimizing target gene codons and conducting genetic breeding research in *Styphnolobium* species. Future studies can carry out comparative analyses of codon usage bias among closely related *Styphnolobium* species, further explore the molecular differentiation characteristics during their evolutionary process, and improve the research system of molecular evolution for the genus *Styphnolobium*.

## Figures and Tables

**Table 1:** Gene List of the Chloroplast Genome of *S. japonicum* f. *oligophyllum*.

Category	Gene Group	Gene Group
Photosynthesis	Subunits of photosystem I	*psaA*, *psaB*, *psaC*, *psaI*, *psaJ*
	Subunits of photosystem II	*psbA*, *psbB*, *psbC*, *psbD*, *psbE*, *psbF*, *psbH*, *psbI*, *psbJ*, *psbK*, *psbL*, *psbM*, *psbN*, *psbT*, *psbZ*
	Subunits of NADH dehydrogenase	ndhA*, ndhB*(2), *ndhC*, *ndhD*, *ndhE*, *ndhF*, *ndhG*, *ndhH*, *ndhI*, *ndhJ*, *ndhK*
	Subunits of cytochrome b/f complex	*petA*, petB*, petD*, *petG*, *petL*, *petN*
	Subunits of ATP synthase	*atpA*, *atpB*, *atpE*, atpF*, *atpH*, *atpI*
	Large subunit of rubisco	*rbcL*
Self-replication	Proteins of large ribosomal subunit	*rpl14*, rpl16*, rpl2*(2), *rpl20*, *rpl23(2)*, *rpl33*, *rpl36*
	Proteins of small ribosomal subunit	*rps11*, rps12*(2), *rps14*, *rps15*, rps16*, *rps18*, *rps19*, *rps2*, *rps3*, *rps4*, *rps7(2)*, *rps8*
	Subunits of RNA polymerase	*rpoA*, *rpoB*, rpoC1*, *rpoC2*
	Ribosomal RNAs	*rrn16S(2)*, *rrn23S(2)*, *rrn4.5S(2)*, *rrn5S(2)*
	Transfer RNAs	trnA-UGC*(2), *trnC-GCA*, *trnD-GUC*, *trnE-UUC*, *trnF-GAA*, *trnG-GCC*, *trnH-GUG*, *trnI-CAU(2)*, trnI-GAU*(2), trnK-UUU*, *trnL-CAA(2)*, trnL-UAA*, *trnL-UAG*, *trnM-CAU*, *trnN-GUU(2)*, *trnP-GGG*, *trnP-UGG*, *trnQ-UUG*, *trnR-ACG(2)*, *trnR-UCU*, *trnS-GCU*, *trnS-GGA*, *trnS-UGA*, trnT-CGU*, *trnT-GGU*, *trnT-UGU*, *trnV-GAC(2)*, trnV-UAC*, *trnW-CCA*, *trnY-GUA*, *trnfM-CAU*
DOther genes	Maturase	*matK*
	Protease	clpP**
	Envelope membrane protein	*cemA*
	Acetyl-CoA carboxylase	*accD*
	c-type cytochrome synthesis gene	*ccsA*
Genes of unknown function	Conserved hypothetical chloroplast ORF	*ycf1(2)*, *ycf2(2)*, ycf3**, *ycf4*

Notes: Gene*: Gene with one introns; Gene**: Gene with two introns; Gene(2): Number of copies of multi-copy genes.

**Table 2:** Summary statistics of codon usage parameters for the 52 protein-coding sequences (CDS) in the chloroplast genome of *S. japonicum* f. *oligophyllum*.

Parameter	Range	Mean ± SD
GC1 (%)	31.79–58.38	46.56 ± 6.75
GC2 (%)	29.07–54.68	39.37 ± 5.50
GC3 (%)	21.69–36.81	28.26 ± 3.36
GCall (%)	29.14–45.1	38.06 ± 4.01
ENC	36.63–52.65	45.40 ± 3.82
CAI	0.10–0.29	0.17 ± 0.04
CBI	−0.22–0.17	−0.10 ± 0.08
Fop	0.27–0.52	0.35 ± 0.05

Note: Detailed values for each of the 52 CDS are provided in Supplementary Table S1.

**Table 3:** Frequency distribution of ENC ratios.

Class Range	Class Mid Value	Number	Frequency
−0.05~0.05	0	13	0.25
0.05~0.15	0.1	27	0.52
0.15~0.25	0.2	11	0.21
0.25~0.35	0.3	1	0.02
Total		52	0.25

## Data Availability

The genome sequence of this study was deposited in GenBank of NCBI (https://www.ncbi.nlm.nih.gov/ (accessed on 10 January 2025)) under accession no. ON571618 (submitted on 20 May 2022, released on 28 June 2022).

## Supplementary Materials

The following supporting information can be downloaded at: https://www.mdpi.com/article/10.3390/cimb48060617/s1.

## References

[1] Arimura S., Nakazato I. (2024). Genome Editing of Plant Mitochondrial and Chloroplast Genomes. Plant Cell Physiol..

[2] Xu C., Dong W., Li W., Lu Y., Xie X., Jin X., Shi J., He K., Suo Z. (2017). Comparative Analysis of Six *Lagerstroemia* Complete Chloroplast Genomes. Front. Plant Sci..

[3] Daniell H., Lin C.-S., Yu M., Chang W.-J. (2016). Chloroplast Genomes: Diversity, Evolution, and Applications in Genetic Engineering. Genome Biol..

[4] Raubeson L.A., Jansen R.K., Henry R.J. (2005). Chloroplast genomes of plants. Plant Diversity and Evolution. Genotypic and Phenotypic Variation in Higher Plants.

[5] Daniell H., Kumar S., Dufourmantel N. (2005). Breakthrough in Chloroplast Genetic Engineering of Agronomically Important Crops. Trends Biotechnol..

[6] Bock R. (2014). Genetic Engineering of the Chloroplast: Novel Tools and New Applications. Curr. Opin. Biotechnol..

[7] Bulle M., Rahman M.M., Kota S., Islam M.R., Keya S.S., Abbagani S., Kirti P.B. (2026). Advancing Chloroplast Bioengineering: Innovations, Regulatory Challenges, and Translational Pathways for Sustainable Agriculture. Int. J. Biol. Macromol..

[8] Ingvarsson P.K. (2008). Molecular Evolution of Synonymous Codon Usage in Populus. BMC Evol. Biol..

[9] Wang J., Qian J., Jiang Y., Chen X., Zheng B., Chen S., Yang F., Xu Z., Duan B. (2022). Comparative Analysis of Chloroplast Genome and New Insights Into Phylogenetic Relationships of *Polygonatum* and Tribe Polygonateae. Front. Plant Sci..

[10] Li C.-L., Zhang P., Cai C.-L., Qin S.-F. (2024). Genomic Characteristics and Codon Preference Analysis of *Schefflera octophylla* (Lour.) Harms Chloroplasts. J. Agric. Sci. Technol..

[11] Geng X., Huang N., Zhu Y., Qin L., Hui L. (2022). Codon Usage Bias Analysis of the Chloroplast Genome of Cassava. S. Afr. J. Bot..

[12] Yao S., Zhang Q., Su M. (2025). Chloroplast Genome Capture History and Genetie Diversity of *Camelia sinensis* Var. *Sinensis* ’Liupao. Guihaia.

[13] Mazumdar P., Binti Othman R., Mebus K., Ramakrishnan N., Ann Harikrishna J. (2017). Codon Usage and Codon Pair Patterns in Non-Grass Monocot Genomes. Ann. Bot..

[14] Hershberg R., Petrov D.A. (2008). Selection on Codon Bias. Annu. Rev. Genet..

[15] Li R., Wang B., Xiao S., Chen L., Yin F., Li J., Jiang C., Zhang D., Zhong Q., Zhang Y. (2025). Characterization of the Complete Chloroplast Genome and Comparative Analysis of the Phylogeny and Codon Usage Bias of Three Yunnan Wild Rice Species. Front. Plant Sci..

[16] Qu Y.-Y., Xin J., Feng F.-F. (2021). Codon Usage Bais in Chloroplast Genome of *Eriobotrya fragrans* Champ. Ex Benth. J. Northwest For. Univ..

[17] Liang Y., Kong Y.-G., Wang Y.-H., Yan Y.-P. (2026). Characteristics and Codon Usage Bias in Chloroplast Genome of *Robinia Neo-Mexicana* Var. *Luxurians*. Cent. South Univ. For. Technol..

[18] Zhao Y. (2007). Study on Taxonomy and Reproduction Characteristics of *Sophora japonica* L. Master’s Thesis.

[19] Mu Z.-Q. (2023). Genetic Diversity of Ancient *Styphnolobium japonicum* Trees in Henan. Master’s Thesis.

[20] Mu Z.-Q., Zhang Y., Zhang B., Cheng Y., Shang F., Wang H. (2023). Intraspecific Chloroplast Genome Variation and Domestication Origins of Major Cultivars of *Styphnolobium japonicum*. Genes.

[21] Bankevich A., Nurk S., Antipov D., Gurevich A.A., Dvorkin M., Kulikov A.S., Lesin V.M., Nikolenko S.I., Pham S., Prjibelski A.D. (2012). SPAdes: A New Genome Assembly Algorithm and Its Applications to Single-Cell Sequencing. J. Comput. Biol..

[22] Kearse M., Moir R., Wilson A., Stones-Havas S., Cheung M., Sturrock S., Buxton S., Cooper A., Markowitz S., Duran C. (2012). Geneious Basic: An Integrated and Extendable Desktop Software Platform for the Organization and Analysis of Sequence Data. Bioinformatics.

[23] Cai Y.-B., Yang X.-Y. (2022). Codon Usage Bias and Its Influencing Factors in the Chloroplast Genome of *Macadamia integrifolia* Maiden & Betche. Plant Sci. J..

[24] Li R.-X., Wang B., Xiao S.-Q. (2025). Assembly, Codon Usage Bias, and Phylogenetic Analysis of Chloroplast Genome of *Oryza meyeriana*. Chin. J. Rice Sci..

[25] Chen W.-Q., Liu Z.-H., Wang M.-J. (2024). Analysis of Codon Usage Bias in Chloroplast Genome of *Halenia elliptica*. Mol. Plant Breed..

[26] Ma Y.-D., Yang Y.-R., Guo C.-C. (2026). Analysis of Codon Bias in Chloroplast Genome of *Cercis Gigantea*. J. Agric. Sci. Technol..

[27] Dai J.-F., Dang L., Zhao X. (2026). Characteristics and Codon Usage Bias in *Laria Principis*-*Rupprechtii* Chloroplast Genome. J. Northwest For. Univ..

[28] Lu Y., Li W.-Q., Xie X.-M. (2018). The Complete Chloroplast Genome Sequence of *Sophora japonica* Var. *Violacea*: Gene Organization and Genomic Resources. Conserv. Genet. Resour..

[29] Shi Y., Liu B. (2020). Complete Chloroplast Genome Sequence of *Sophora japonica* ‘JinhuaiJ2’ (Papilionaceae), an Important Traditional Chinese Herb. Mitochondrial DNA B Resour..

[30] Liao M., Gao X.-F., Zhang J.-Y., Deng H.-N., Xu B. (2021). Comparative Chloroplast Genomics of Sophora Species: Evolution and Phylogenetic Relationships in the Early-Diverging Legume Subfamily Papilionoideae (Fabaceae). Front. Plant Sci..

[31] Duan N., Ru D., Liu B. (2025). Comparative chloroplast genomes of *Sophora* species: Identification of variable DNA markers and phylogenetic relationships within the genus. BMC Plant Biol..

[32] Rang Z.C., Hu X.-Y., Liu Y.-P., Su X., Liu T., Mao X.-R., Xu Y.-J., Yang P., Zhang P.-H., Zheng C.-Y. (2025). Codon usage bias and phylogenetic analysis of chloroplast genome in *Sophora alopecuroides* (Fabaceae). Guihaia.

[33] Bhattacharyya D., Uddin A., Das S., Chakraborty S. (2019). Mutation Pressure and Natural Selection on Codon Usage in Chloroplast Genes of Two Species in *Pisum* L. (Fabaceae: Faboideae). Mitochondrial DNA A.

[34] Mu J.-J., Zhang J.-S. (2025). Codon Usage Bias Analysis in the Chloroplast Genome of *Actinostemma tenerum* (Cucurbitaceae). Curr. Issues Mol. Biol..

[35] Liang X.-L., Guo S. (2022). Codon Usage Bias in the Chloroplast Genome of *Sphaerophysa salsula*. J. Northwest For. Univ..

[36] Xiao M.-K., Nie K.-H., Shen Z.-B. (2023). Analysis of Codon Usage Bias in the Chloroplast Genome of *Koelreuteria bipinnata*. J. Southwest For. Univ..

[37] Zheng M., Zhao Y., Gao M., Huang M., Song X. (2026). Chloroplast structure, codon usage bias, and machine learning-based molecular identification using DNA barcoding of Sophorae Tonkinensis Radix et Rhizoma (Shan Dou Gen) and its analogues. Fitoterapia.

