# Supplementary material for: Analysis of Chloroplast Genome Characteristics and Codon Usage Bias of Styphnolobium japonicum f. oligophyllum
Source: Curr Issues Mol Biol. 2026 Jun 15;48(6):617. doi: 10.3390/cimb48060617 (PMC13298257; doi:10.3390/cimb48060617)
Supplement: Supplementary file 1 [file cimb-48-00617-s001.zip › cimb-4311422-supplementary.pdf]

Supplementary files

Tables legends

Table S1.Codon GC content and main codon usage bias parameters in chloroplast genome of *S. japonicum* f. *oligophyllum*.

Table S2. Identification of optimal codons in the chloroplast genome of *S. japonicum* f. *oligophyllum*.

Table S3. Key codon usage bias parameters of chloroplast genomes from cited references

Table S1. Codon GC content and main codon usage bias parameters in chloroplast genome of *S. japonicum* f. *oligophyllum*.

Genes	GC content/%				ENC	CAI	CBI	Fop
	GC1	GC2	GC3	GCall				
accD	38.63	35.81	28.77	34.41	41.08	0.20	-0.18	0.34
atpA	54.99	40.12	26.03	40.38	46.40	0.21	-0.02	0.41
atpB	56.71	41.68	27.25	41.88	46.76	0.20	0.00	0.41
atpE	50.75	39.55	27.61	39.30	44.09	0.15	-0.17	0.31
atpF	44.97	34.92	33.86	37.92	42.82	0.14	-0.13	0.34
atpI	48.79	35.48	23.79	36.02	38.43	0.20	0.01	0.40
ccsA	31.79	37.35	25.00	31.38	42.36	0.14	-0.22	0.29
cemA	39.13	29.13	32.17	33.48	48.05	0.20	-0.02	0.40
clpP	58.38	36.55	30.96	41.96	50.55	0.18	-0.09	0.35
matK	37.72	30.26	27.50	31.83	47.44	0.16	-0.18	0.31
ndhA	42.31	38.46	21.70	34.16	40.47	0.14	-0.10	0.33
ndhB	42.19	39.55	31.03	37.59	46.19	0.16	-0.08	0.36
ndhC	46.28	33.88	24.79	34.99	47.40	0.17	-0.15	0.31
ndhD	38.20	37.40	29.40	35.00	45.40	0.14	-0.12	0.33
ndhE	40.20	34.31	28.43	34.31	44.04	0.16	-0.12	0.33
ndhF	36.28	34.67	21.69	30.88	41.52	0.15	-0.20	0.29
ndhG	45.20	34.46	23.73	34.46	44.38	0.14	-0.20	0.27
ndhH	52.54	35.28	25.63	37.82	49.10	0.17	-0.09	0.35
ndhI	43.83	35.80	22.84	34.16	47.31	0.21	-0.13	0.34
ndhJ	50.31	36.48	27.67	38.16	38.97	0.16	-0.17	0.31
ndhK	44.20	44.64	29.91	39.58	49.20	0.16	-0.17	0.32
petA	53.44	36.88	30.00	40.10	47.60	0.18	-0.10	0.35
petB	47.22	41.67	30.09	39.66	40.90	0.22	-0.06	0.37
petD	51.55	39.13	26.71	39.13	38.30	0.15	-0.18	0.27
psaA	52.33	43.28	32.09	42.57	48.90	0.19	-0.10	0.36
psaB	48.98	43.27	31.16	41.13	47.19	0.18	-0.10	0.36
psbA	50.00	43.22	32.77	42.00	40.67	0.29	0.17	0.52
psbB	55.01	46.37	30.06	43.81	43.11	0.18	-0.08	0.37
psbC	53.38	45.99	30.59	43.32	45.80	0.18	-0.02	0.40
psbD	52.26	43.22	33.62	43.03	44.70	0.26	0.08	0.46
rbcL	57.56	43.69	30.23	43.82	47.15	0.26	0.06	0.46
rpl2	50.72	48.55	31.88	43.72	51.11	0.14	-0.12	0.35

rpl14	51.22	37.40	27.64	38.75	48.92	0.17	-0.09	0.35
rpl16	50.00	51.47	30.88	44.12	45.34	0.11	-0.10	0.36
rpl20	35.29	38.66	26.89	33.61	47.63	0.10	-0.18	0.30
rpoA	45.48	31.02	23.80	33.43	41.45	0.15	-0.13	0.33
rpoB	49.95	38.47	28.48	38.97	47.08	0.15	-0.12	0.34
rpoC1	49.64	37.37	27.74	38.25	48.49	0.16	-0.12	0.34
rpoC2	44.64	36.27	27.75	36.22	47.16	0.15	-0.15	0.33
rps2	43.46	43.46	27.85	38.26	48.65	0.17	-0.15	0.33
rps3	45.66	33.79	23.74	34.40	47.54	0.16	-0.12	0.35
rps4	51.49	38.12	25.25	38.28	46.54	0.17	0.03	0.42
rps7	52.56	45.51	26.28	41.45	45.62	0.19	-0.07	0.38
rps8	37.04	41.48	26.67	35.06	39.88	0.11	-0.04	0.38
rps11	53.24	54.68	25.90	44.60	48.66	0.15	-0.14	0.34
rps12	52.94	49.58	32.77	45.10	48.37	0.14	-0.05	0.37
rps14	43.56	46.53	29.70	39.93	37.80	0.12	-0.17	0.31
rps18	33.06	38.71	25.00	32.26	36.63	0.16	-0.04	0.40
ycf1	33.72	29.07	24.64	29.14	45.23	0.16	-0.15	0.34
ycf2	41.47	34.52	36.81	37.60	50.91	0.15	-0.14	0.34
ycf3	48.52	38.46	31.36	39.45	52.65	0.16	-0.16	0.35
ycf4	42.16	41.62	31.35	38.38	48.65	0.16	-0.07	0.36
Average Mean	46.56	39.37	28.26	38.06	45.40	0.17	-0.10	0.35

Table S2. Identification of optimal codons in the chloroplast genome of *S. japonicum* f. *oligophyllu*

AA	Codon	High expression		Low expression		ΔRSCU	AA	Codon	High expression		Low expression		ΔRSCU
		Number	RSCU	Number	RSCU				Number	RSCU	Number	RSCU	
Ala	GCA	13	1.13	29	1.06	0.07	Leu	CUA	8	0.49	48	0.9	-0.41
	GCC	6	0.52	25	0.92	-0.4		CUC	2	0.12	30	0.56	-0.44
	GCG	5	0.43	13	0.48	-0.05		CUG	3	0.18	30	0.56	-0.38
	**GCU****	22	1.91	42	1.54	0.37		CUU	23	1.41	78	1.46	-0.05
Arg	AGA	11	1.47	62	1.75	-0.28		**UUA*****	42	2.57	58	1.08	1.49
	AGG	6	0.8	34	0.96	-0.16		UUG	20	1.22	77	1.44	-0.22
	CGA	11	1.47	52	1.46	0.01	Lys	**AAA****	42	1.71	125	1.34	0.37
	CGC	2	0.27	17	0.48	-0.21		AAG	7	0.29	62	0.66	-0.37
	CGG	0	0	21	0.59	-0.59	Met	AUG	15	1	65	1	0
	**CGU*****	15	2	27	0.76	1.24	Phe	UUC	15	0.7	87	0.94	-0.24
Asn	AAC	5	0.36	45	0.49	-0.13		**UUU***	28	1.3	99	1.06	0.24
	**AAU***	23	1.64	137	1.51	0.13	Pro	CCA	12	1	32	0.96	0.04
Asp	GAC	3	0.25	34	0.41	-0.16		**CCC****	15	1.25	28	0.84	0.41
	**GAU***	21	1.75	132	1.59	0.16		CCG	6	0.5	24	0.72	-0.22
Cys	UGC	1	0.33	12	0.59	-0.26		CCU	15	1.25	50	1.49	-0.24

	**UGU***	5	1.67	29	1.41	0.26	Ser	AGC*	4	0.43	13	0.26	0.17
Gln	**CAA***	24	1.66	85	1.44	0.22		**AGU***	12	1.29	52	1.05	0.24
	CAG	5	0.34	33	0.56	-0.22		UCA	10	1.07	63	1.28	-0.21
Glu	**GAA***	27	1.46	112	1.24	0.22		UCC	7	0.75	55	1.11	-0.36
	GAG	10	0.54	69	0.76	-0.22		UCG*	7	0.75	33	0.67	0.08
Gly	GGA	20	1.67	71	1.69	-0.02		**UCU***	16	1.71	80	1.62	0.09
	GGC	2	0.17	19	0.45	-0.28	Thr	ACA	12	1.3	49	1.41	-0.11
	GGG	6	0.5	40	0.95	-0.45		ACC	7	0.76	27	0.78	-0.02
	**GGU*****	20	1.67	38	0.9	0.77		ACG	3	0.32	19	0.55	-0.23
His	CAC	2	0.24	17	0.45	-0.21		**ACU****	15	1.62	44	1.27	0.35
	**CAU***	15	1.76	58	1.55	0.21	Trp	UGG	16	1	56	1	0
Ile	AUA	20	0.97	89	1.07	-0.1	Val	**GUA***	22	1.57	41	1.31	0.26
	AUC	9	0.44	57	0.69	-0.25		GUC	6	0.43	17	0.54	-0.11
	**AUU****	33	1.6	103	1.24	0.36		GUG	7	0.5	23	0.74	-0.24
Tyr	UAC	5	0.36	22	0.35	0.01		**GUU***	21	1.5	44	1.41	0.09
	UAU	23	1.64	102	1.65	-0.01							

Note: "*" indicates ΔRSCU ≥ 0.08, "**" indicates ΔRSCU ≥ 0.3, "***" indicates ΔRSCU ≥ 0.5;underlined codons represent high-frequency codons with RSCU > 1; bold codons represent optimal codons.

Table S3. Key codon usage bias parameters of chloroplast genomes from cited references

Species	Mean ENC	GC3 content/%	Number of optimal codons	Dominant driving force
Styphnolobium. japonicum f. *oligophyllum*	45.4	28.26	19	natural
Actinostemma tenerum	45.57	28.12	18	natural
Sophorae Tonkinensis	/	/	/	natural
Sophora alopecuroides	/	36.6	21	natural
Koelreuteria bipinnata	/	29.84	12	natural
Sphaerophysa salsula	47.11	/	16	Mutation + natural
Eriobotrya fragrans	47.02	28.44	15	Mutation + natural selection
Macadamia integrifolia	48.8	29.72	16	Mutation + natural
